# Analysis of *Staphylococcus aureus* infections among pediatric Indian patients

**DOI:** 10.6026/9732063002001275

**Published:** 2024-10-31

**Authors:** Priyank Rajan, Nayan Chaudhary, Mukesh Sanklecha, Mayur Wanjari, Labdhi Sangoi, Ravi Sangoi

**Affiliations:** 1 Clinical Fellow in Paediatric Haematology Oncology and BMT, Bristol Royal Hospital for Children, Bristol, United Kingdom; 2 Department of Paediatrics, Bombay Hospital and Institute of Medical Sciences, Mumbai, India; 3 Consultant Paediatrician, Bombay Hospital and Institute of Medical Sciences, Mumbai, India; 4 Department of Research, Datta Meghe Institute of Higher Education & Research (DMIHER), Sawangi, Maharashtra, India; 5 Department of Research, Government Medical College, Jalna, Maharashtra; 6 Department of Internal Medicine, Government Medical College and General Hospital, Baramati, Pune, Maharashtra, India

**Keywords:** Staphylococcus aurous, MRSA, MSSA, infections, colonization patterns, antibiotic resistance, vancomycin, teicoplanin, daptomycin, tertiary care, nasal carriage, antibiotic susceptibility

## Abstract

*Staphylococcus aureus* is a leading cause of infections in paediatric populations, ranging from mild skin infections to life-threatening systemic infections. Methicillin-resistant *Staphylococcus aureus* [MRSA] has become increasingly prevalent, raising concerns regarding treatment and control measures. Therefore, it is of interest to assess the *Staphylococcus aureus* infections in hospitalized children and determine the colonization patterns and antibiotic sensitivity profiles at a tertiary care centre. A prospective observational study was conducted on 103 paediatric patients, categorized into *S. aureus*-infected and healthy controls. *S. aureus* isolates were obtained from clinical specimens and colonization was assessed using nasal, axillary, throat and inguinal swabs. Antibiotic susceptibility was tested using the MIC method. *S. aureus* infection was confirmed in 32 [31.06%] of the patients, with colonization observed in 71.87% of infected cases. Among the colonized sites, nasal and axillary regions were the most frequent. MRSA accounted for 46.8% of the infections, while MSSA made up 53.2%. MRSA isolates were more resistant to antibiotics compared to MSSA. Vancomycin, daptomycin and teicoplanin showed 100% efficacy against both MRSA and MSSA. Colonization was significantly higher in infected patients compared to controls, indicating colonization as a risk factor for *S. aureus* infection. Antibiotic sensitivity patterns suggest that vancomycin and teicoplanin remain effective against MRSA, but increasing resistance underscores the need for careful antibiotic selection and preventive measures in paediatric care.

## Background:

*Staphylococcus aureus* is a highly versatile gram-positive bacterium, commonly responsible for an extensive array of infections, from relatively benign cutaneous and soft tissue infections to life-threatening diseases, such as pneumonia, endocarditis, osteomyelitis and bacteraemia [1]. The pathogen is considered to be a leading cause of morbidity in communities and hospitals and is quite challenging, considering the fact that it is capable of developing resistance to many antibiotics [2]. The emergence of MRSA has indeed made the management of these infections more challenging, with few efficacious treatments available that necessitate repeated therapy with toxic or substandard antibiotics [3]. Many countries have reported an increased morbidity and mortality incidence owing to this organism, alone or in concurrence with other disease-causing agents, especially amid 'frail' populations such as children [4]. In children infections with *Staphylococcus aureus* pose a high burden in selected populations, especially those with increased susceptibility due to an underlying condition or compromised immune systems [5]. For example, patients in the PICU are at an increased risk for MRSA colonization and infections. The extensive use of invasive devices, longer lengths of hospital stay and exposure to a wide spectrum of antibiotic agents largely contribute to this emerging problem [6]. Studies have identified that even in children; MRSA infection rates are on a rise in most countries, including developing countries such as India, where nosocomial infection is a cause for concern [7]. Many research studies have identified colonization by *Staphylococcus aureus*, particularly from the nares, as one of the important risk factors for development of infection [8]. It is estimated that 20-30% of the population are colonized with *Staphylococcus aureus*, although the risk of progression from colonization to infection is higher in children [9]. Invasive infections have been highly associated with nasal colonization by MRSA. To prevent these infections, decolonization strategies involving mupirocin and chlorhexidine have been commonly practiced in high risk areas [10]. Therefore, epidemiologically, MRSA has undergone modification with time and now two major variants of the bacterium: hospital acquired [HA] MRSA and community acquired [CA] MRSA [11]. HA-MRSA causes much more virulent infections in previously exposed subjects like those who have been recently admitted to PICUs, while CA-MRSA has emerged as a leading pathogen even in healthy individuals with no recent hospital contact [12]. Where CA-MRSA tends to cause skin and soft tissue infection, other severe infections, including pneumonia associated with the use of a ventilator and invasive infection of the blood, are usually caused by HA-MRSA [13]. For both MRSA and MSSA infections, even among younger children, there has been an increase; therefore, local epidemiologic data on resistance patterns should be considered in choosing appropriate therapy for effective treatment. Recent literature has demonstrated that active surveillance and colonization are the main protagonists of reducing the transmission of MRSA within tertiary care institutions among children [14, 15]. This study aims to analyse the spectrum of *S. aureus* infections in paediatric patients admitted to a tertiary care centre, focusing on colonization trends, antibiotic resistance patterns, and treatment implications.

## Materials and Methods:

A prospective observational study was conducted from 2016 to 2018 in the paediatric department of a tertiary care hospital in Mumbai, India, a referral centre for paediatric care serving a large population with diverse healthcare needs. The Institutional Review Board obtained ethical approval, and the legal guardians of all participants provided informed consent prior to their inclusion in the study. The study included hospitalized children aged 0 to 18 years, whose guardians gave their consent. We recruited both patients with symptoms related to infection and those undergoing routine admissions for unrelated medical conditions, serving as the infected and control groups, respectively. The exclusion criteria included children treated as outpatients, those discharged before completing full diagnostic evaluations, and those whose guardians declined to participate. A power analysis, based on published studies on *Staphylococcus aureus* colonization and infection rates in paediatric patients, was conducted to determine an adequate sample size. We calculated a minimum sample size of 100 patients with an estimated *S. aureus* infection prevalence of 30%, a two-tailed hypothesis, a confidence level of 0.05 and a power of 80%. This analysis assumed a clinically relevant 20% difference in colonization rates between infected and control groups. A final sample size of 103 children were included in the study, comprising 32 patients with confirmed *S. aureus* infections and 71 control patients without infections.

We used sterile, wet cotton swabs to get samples from the nasal passages, axillae, throat and inguinal region of all 103 patients to test for *S. aureus* colonization. We obtained additional bacterial cultures from infected sites, such as blood, pus and other sterile body fluids, when we suspected systemic infections or abscesses. We promptly transported all samples to the microbiology laboratory for analysis. We processed the specimens using standard microbiological procedures. We grew the cultures on blood agar and MacConkey agar plates and incubated them at 37°C for 24 to 48 hours. We used colony morphology and Gram staining to identify Gram-positive cocci arranged in clusters. We used the catalase and coagulase tests to confirm that the isolates that were positive for coagulase were indeed *S. aureus*. To find out if the bacteria were resistant to methicillin, they were tested for susceptibility to cefoxitin [30 µg] using the disk diffusion method, which is recommended by the Clinical and Laboratory Standards Institute [CLSI]. We performed antibiotic susceptibility testing using the Minimum Inhibitory Concentration [MIC] method with the BD Phoenix automated system. Antibiotics tested included vancomycin, teicoplanin, daptomycin, clindamycin, ciprofloxacin and trimethoprim-sulfamethoxazole. We found Methicillin-resistant *S. aureus* [MRSA] when isolates were resistant to cefoxitin, which showed that the mecA gene was present. We statistically analysed the collected data using SPSS version 22.0.

## Results:

## Gender-wise distribution of infected patients:

In this study, the prevalence of *Staphylococcus aureus* infections was observed to be higher among male paediatric patients than females. Out of the 32 infected patients, 22 [68.75%] were male, while only 10 [31.25%] were female. This finding suggests a possible higher susceptibility to *S. aureus* infections among male children in the study population as shown in Table 1.

## Age-wise distribution of infected patients:

The age-wise distribution of *S. aureus* infections revealed that children in the 0-5 year's age group were the most affected. Among the 32 infected patients, 12 [37.5%] were aged 0-5 years, 10 [31.25%] were in the 6-10 years age group, and another 10 [31.25%] were in the 11-18 years age group as shown in Table 2.

## Primary sites of infection:

The most common site of *S. aureus* isolation was from pus, which accounted for 18 [56.25%] of the 32 infected patients. Blood and joint effusion were also notable infection sites, both with 4 cases [12.5%], while catheter tips were implicated in 2 [6.25%] cases. These results indicate that skin and soft tissue infections were the most prevalent in this cohort as shown in Table 3.

Figure 1 - see PDF illustrates the distribution of various types of *Staphylococcus aureus* infections among the children. Figure 2 - see PDF shows a comparative bar chart illustrating the resistance patterns of MRSA and MSSA isolates to various antibiotics. Figure 3 - see PDF shows a stacked bar chart comparing clinical outcomes between MRSA and MSSA infections. Figure 4 - see PDF shows a pie chart demonstrating the proportion of hospital-acquired versus community-acquired infections.

## Discussion:

The high prevalence of MRSA infection reported in this study of 46.8% matches global trends; global trends indicate a rising trend in the culture prevalence of MRSA in both health care and community settings [16]. These findings highlight critical knowledge about colonization trends, resistance to antibiotics and their impact on clinical outcomes in the successful treatment of such infections. For a long time, experts have recognized nasal and cutaneous colonization with *S. aureus* as a predisposing factor for infection, particularly in immune compromised or hospitalized patients [17]. Colonization was significantly higher in the MRSA-infected group than in the control group, demonstrating that colonization is definitely one of the most important risks for subsequent infection. Especially, association of nasal cavities colonized with invasive *S. aureus* infections has already been reported in both paediatric and adult groups [18]. In a few studies, it was recently indicated that children with colonization by S. *Staphylococcus aureus* become more resistant to infection, especially in intensive care settings where the use of invasive medical devices and prolongation of hospital confinement heightens susceptibility to the spread of the microorganism [19]. However, there is an effective decolonization treatment with mupirocin nasal ointment application and chlorhexidine body baths that proves to reduce MRSA colonization and the rates of infection associated with it among high-risk populations [20]. Global findings further support the resistive features of antibiotics presented in the current study, as they indicate an increasing trend of resistance in MRSA bacteria. In the studied population, MRSA strains had a higher prevalence of resistance to common antibiotics such as clindamycin, ciprofloxacin, and trimethoprim-sulfamethoxazole. These findings are in agreement with recent reports showing an increase in MRSA resistance against various antibiotic classes, which limits and further complicates the therapeutic options to treat such infections [21]. Of even greater concern, the resistance exhibited to clindamycin is of particular interest since it is often prescribed as an alternative when patients present allergies against β-lactam antibiotics [22]. This high incidence of resistance to ciprofloxacin in this study is very alarming and stresses the judicious use of antibiotics to prevent further resistance, considering the broad use of this antibiotic against a range of infections [23]. Regarding susceptibility, both MRSA and MSSA were 100% susceptible to vancomycin, teicoplanin, and daptomycin, but their over-reliance on vancomycin is difficult due to its well-known nephrotoxicity, especially in the paediatric population [24]. Recent publications describing VISA and VRSA have further complicated the situation, raising questions about the future effectiveness of vancomycin in treating MRSA infections [25]. Although reports of VRSA remain rare, increased use of vancomycin has introduced selective factors in favour of developing resistant strains [26], which will likely increase the urgent need for either alternative treatment modalities or new antibiotic discovery focused on the treatment of resistant strains. Another study found daptomycin and teicoplanin to be valuable alternatives in this regard. Economic reasons have significantly contributed to their relatively restricted use. A larger study on clinical profile of Staphylococcus infection in pediatrics is urgently needed to define the exact magnitude of the problem [27- 28].

## Conclusion:

*Staphylococcus aureus* continues to be a significant pathogen in paediatric populations, with colonization proving to be a critical risk factor for subsequent infections, particularly in hospitalized and immune compromised children. The high prevalence of Methicillin-resistant *Staphylococcus aureus* [MRSA] observed in this study underscores the urgent need for robust infection control measures. We should routinely implement screening and decolonization strategies, especially in high-risk environments like intensive care units, to prevent the spread of MRSA.

## Figures and Tables

**Table 1:** Gender-wise distribution of infected patients

**Gender**	**Infected patients [n=32]**	**Percentage**
Male	22	68.75%
Female	10	31.25%

**Table 2:** Age-wise distribution of infected patients

**Gender**	**Infected patients [n=32]**	**Percentage**
Male	22	68.75%
Female	10	31.25%

**Table 3:** Primary sites of infection in patients

**Sites**	**Infected patients [n=32]**	**Percentage**
Pus	18	56.25%
Blood	4	12.50%
